# (1*S*,3*R*,8*R*)-2,2-Dibromo-3,7,7,10-tetra­methyltricyclo­[6.4.0.0^1,3^]dodec-9-en-11-one

**DOI:** 10.1107/S1600536812046089

**Published:** 2012-11-14

**Authors:** Abdelouahd Oukhrib, Ahmed Benharref, Mohamed Saadi, Lahcen El Ammari, Moha Berraho

**Affiliations:** a Laboratoire de Chimie des Substances Naturelles, "Unité Associé au CNRST (URAC16)", Faculté des Sciences Semlalia, BP 2390 Bd My Abdellah, 40000 Marrakech, Morocco; b Laboratoire de Chimie du Solide Appliquée, Faculté des Sciences, Avenue Ibn Battouta, BP 1014 Rabat, Morocco

## Abstract

The title compound, C_16_H_22_Br_2_O, was synthesized from β-himachalene (3,5,5,9-tetra­methyl-2,4a,5,6,7,8-hexa­hydro-1*H*-benzocyclo­heptene), which was isolated from the essential oil of the Atlas cedar (*Cedrus Atlantica*). The mol­ecule is built up from fused six- and seven-membered rings and an additional three-membered ring from the reaction of himachalene with dibromo­carbene. The six-membered ring has an envelope conformation, with the C atom belonging to the three-membered ring forming the flap, whereas the seven-membered ring displays a screw-boat conformation; the dihedral angle between the rings (all atoms) is 60.92 (16)°.

## Related literature

For the isolation of β-himachalene, see: Joseph & Dev (1968 ▶); Plattier & Teiseire (1974 ▶). For the reactivity of this sesquiterpene, see: Lassaba *et al.* (1997 ▶); Chekroun *et al.* (2000 ▶); El Jamili *et al.* (2002 ▶); Sbai *et al.* (2002 ▶); Dakir *et al.* (2004 ▶). For its biological activity, see: Daoubi *et al.* (2004 ▶). For conformational analysis, see: Cremer & Pople (1975 ▶).
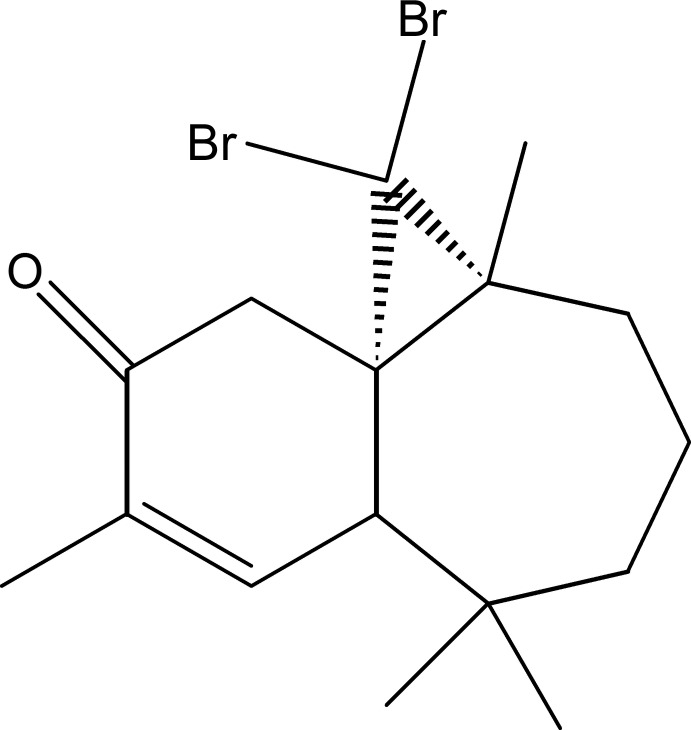


## Experimental

### 

#### Crystal data

C_16_H_22_Br_2_O
*M* *_r_* = 390.16
Orthorhombic, 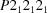

*a* = 6.7369 (1) Å
*b* = 14.7635 (3) Å
*c* = 16.3543 (3) Å
*V* = 1626.60 (5) Å^3^
*Z* = 4
Mo *K*α radiation
μ = 4.98 mm^−1^
*T* = 298 K
0.80 × 0.65 × 0.25 mm

#### Data collection

Bruker APEXII CCD diffractometer
Absorption correction: multi-scan (*SADABS*; Sheldrick, 2003 ▶) *T*_min_ = 0.259, *T*_max_ = 0.746
11891 measured reflections
3330 independent reflections
2939 reflections with *I* > 2σ(*I*)
*R*_int_ = 0.028

#### Refinement

*R*[*F* ^2^ > 2σ(*F* ^2^)] = 0.030
*wR*(*F* ^2^) = 0.074
*S* = 1.06
3330 reflections
176 parameters
H-atom parameters constrained
Δρ_max_ = 0.68 e Å^−3^
Δρ_min_ = −0.37 e Å^−3^
Absolute structure: Flack (1983) ▶, 1397 Friedel pairs
Flack parameter: 0.021 (12)

### 

Data collection: *APEX2* (Bruker, 2009 ▶); cell refinement: *SAINT* (Bruker, 2009 ▶); data reduction: *SAINT*; program(s) used to solve structure: *SHELXS97* (Sheldrick, 2008 ▶); program(s) used to refine structure: *SHELXL97* (Sheldrick, 2008 ▶); molecular graphics: *ORTEP-3 for Windows* (Farrugia, 2012 ▶); software used to prepare material for publication: *WinGX* (Farrugia, 2012 ▶).

## Supplementary Material

Crystal structure: contains datablock(s) I, global. DOI: 10.1107/S1600536812046089/go2074sup1.cif

Structure factors: contains datablock(s) I. DOI: 10.1107/S1600536812046089/go2074Isup2.hkl

Supplementary material file. DOI: 10.1107/S1600536812046089/go2074Isup3.cml

Additional supplementary materials:  crystallographic information; 3D view; checkCIF report

## Figures and Tables

**Fig. 1.**
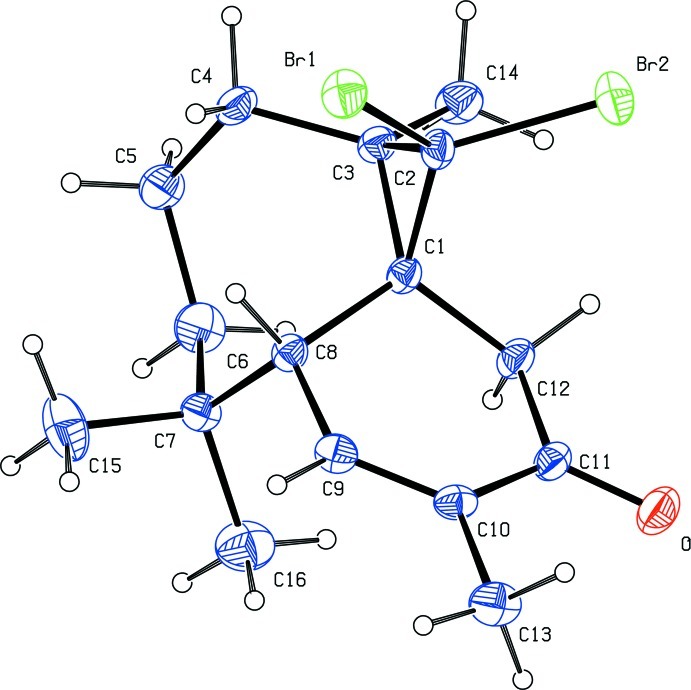
: Molecular structure of the title compound with the atom-labelling scheme. Displacement ellipsoids are drawn at the 30% probability. level. H atoms are represented as small spheres of arbitrary radii.

## supplementary crystallographic information

### Comment

The essential oil of the Atlas cedar (Cedrus atlantica) consist mainly (50%) of a bicyclic hydrocarbon called β-himachalene (Plattier & Teiseire(1974); Joseph & Dev (1968)). The reactivity of this sesquiterpene and its derivatives has been studied extensively by our team in order to prepare new products having biological proprieties. (Lassaba *et al.*, 1997; Chekroun *et al.*, 2000; El Jamili *et al.*, 2002; Sbai *et al.*, 2002; Dakir *et al.*, 2004). Indeed, these compounds were tested, using the food poisoning technique, for their potential antifungal activity against the phytopathogen Botrytis cinerea (Daoubi *et al.*, 2004). In a previous work (El Jamili *et al.*, 2002), we have prepared, from β-himachalene, the (1*S*,3*R*,8*R*)-2,2-dibromo- 3,7,7,10- tetramethyltricyclo [6.4.0.01,3] dodec-9-ene, which is treated with *N*-bromosuccinimide and gave the title compound. The structure of this new product was determined by its single-crystal X-ray structure. The molecule is built up from two fused six-and seven- membered rings and an additional three-membered ring from the reaction with the carbene (Fig.1). The six-membered ring has an envelope conformation, as indicated by the total puckering amplitude QT = 0.433 (3) Å and spherical polar angle θ= 123.1 (4)° with φ = 181.6 (5)°, whereas the seven-membered ring display a screw boat conformation with QT = 1.1208 (4) Å, θ = 88.14 (2)°, φ2 = -49.94 (2)° and φ3 = -93.26 (5)° (Cremer & Pople, 1975). Owing to the presence of Br atoms, the absolute configuration could be fully confirmed, by refining the Flack parameter (Flack & Bernardinelli (2000)) as C1(S), C3(*R*) and C8(*R*).

### Experimental

In a reactor containing a solution of (1*S*, 3*R*, 8*R*)-2,2-dibromo- 3,7,7,10 tetramethyltricyclo [6.4.0.01,3] dodec-9-ene (1 g, 2,6 mmol) in 50 ml of tetrahydrofuran and water (THF/H2O) (4:1) cooled to 273 K and kept in the dark, was added in small portions 1 g (5,6 mmol) of *N*-bromosccinimide. The reaction mixture was left stirring for 1 h, after which 20 ml of a saturated solution of NaHCO_3_ was added. Subsequently, the extraction was performed three times with diethyl ether (3 *x* 20 ml). The organic extracts were dried over Na_2_S_O_4, filtered, concentrated, and chromatographed. The title compound was obtained with a yield of 80% and was recrystallized in n-pentane.

### Refinement

All H atoms were fixed geometrically and treated as riding with C—H = 0.96 Å (methyl),0.97 Å (methylene), 0.98 Å (methine) with *U*_iso_(H) = 1.2Ueq(methylene, methine) or *U*_iso_(H) = 1.5Ueq(methyl).

### Crystal data

**Table:**

C_16_H_22_Br_2_O	*F*(000) = 784
*M**_r_* = 390.16	*D*_x_ = 1.593 Mg m^−^^3^
Orthorhombic, *P*2_1_2_1_2_1_	Mo *K*α radiation, λ = 0.71073 Å
Hall symbol: P 2ac 2ab	Cell parameters from 11891 reflections
*a* = 6.7369 (1) Å	θ = 2.8–26.4°
*b* = 14.7635 (3) Å	µ = 4.98 mm^−^^1^
*c* = 16.3543 (3) Å	*T* = 298 K
*V* = 1626.60 (5) Å^3^	Prism, colourless
*Z* = 4	0.80 × 0.65 × 0.25 mm

### Data collection

**Table:**

Bruker APEXII CCD diffractometer	3330 independent reflections
Radiation source: fine-focus sealed tube	2939 reflections with *I* > 2σ(*I*)
Graphite monochromator	*R*_int_ = 0.028
ω and φ scans	θ_max_ = 26.4°, θ_min_ = 2.9°
Absorption correction: multi-scan (*SADABS*; Sheldrick, 2003)	*h* = −7→8
*T*_min_ = 0.259, *T*_max_ = 0.746	*k* = −18→18
11891 measured reflections	*l* = −20→20

### Refinement

**Table:**

Refinement on *F*^2^	Secondary atom site location: difference Fourier map
Least-squares matrix: full	Hydrogen site location: inferred from neighbouring sites
*R*[*F*^2^ > 2σ(*F*^2^)] = 0.030	H-atom parameters constrained
*wR*(*F*^2^) = 0.074	*w* = 1/[σ^2^(*F*_o_^2^) + (0.034*P*)^2^ + 0.368*P*] where *P* = (*F*_o_^2^ + 2*F*_c_^2^)/3
*S* = 1.06	(Δ/σ)_max_ = 0.001
3330 reflections	Δρ_max_ = 0.68 e Å^−^^3^
176 parameters	Δρ_min_ = −0.37 e Å^−^^3^
0 restraints	Absolute structure: Flack (1983), 1397 Friedel pairs
Primary atom site location: structure-invariant direct methods	Flack parameter: 0.021 (12)

### Special details

**Table:**

Geometry. All s.u.'s (except the s.u. in the dihedral angle between two l.s. planes) are estimated using the full covariance matrix. The cell s.u.'s are taken into account individually in the estimation of s.u.'s in distances, angles and torsion angles; correlations between s.u.'s in cell parameters are only used when they are defined by crystal symmetry. An approximate (isotropic) treatment of cell s.u.'s is used for estimating s.u.'s involving l.s. planes.
Refinement. Refinement of *F*^2^ against ALL reflections. The weighted *R*-factor *wR* and goodness of fit *S* are based on *F*^2^, conventional *R*-factors *R* are based on *F*, with *F* set to zero for negative *F*^2^. The threshold expression of *F*^2^ > 2σ(*F*^2^) is used only for calculating *R*-factors(gt) *etc*. and is not relevant to the choice of reflections for refinement. *R*-factors based on *F*^2^ are statistically about twice as large as those based on *F*, and *R*- factors based on ALL data will be even larger.

### Fractional atomic coordinates and isotropic or equivalent isotropic displacement parameters (Å^2^)

**Table:**

	*x*	*y*	*z*	*U*_iso_*/*U*_eq_	
C1	0.7886 (4)	0.8949 (2)	0.72986 (18)	0.0345 (7)	
C2	0.7652 (4)	0.8664 (2)	0.8186 (2)	0.0381 (7)	
C3	0.8277 (5)	0.9620 (2)	0.79993 (18)	0.0381 (7)	
C4	0.6771 (5)	1.0379 (2)	0.8104 (2)	0.0458 (8)	
H4A	0.7011	1.0681	0.8621	0.055*	
H4B	0.5449	1.0120	0.8124	0.055*	
C5	0.6854 (6)	1.1078 (2)	0.7417 (2)	0.0545 (9)	
H5A	0.5580	1.1383	0.7392	0.065*	
H5B	0.7842	1.1529	0.7560	0.065*	
C6	0.7335 (7)	1.0720 (3)	0.6570 (3)	0.0635 (11)	
H6A	0.8704	1.0516	0.6578	0.076*	
H6B	0.7270	1.1228	0.6195	0.076*	
C7	0.6078 (5)	0.9950 (2)	0.62020 (19)	0.0411 (7)	
C8	0.6031 (4)	0.9087 (2)	0.67808 (18)	0.0353 (7)	
H8	0.4927	0.9177	0.7163	0.042*	
C9	0.5589 (4)	0.8232 (2)	0.63150 (19)	0.0417 (7)	
H9	0.4286	0.8142	0.6145	0.050*	
C10	0.6912 (5)	0.7587 (2)	0.61230 (18)	0.0391 (7)	
C11	0.9012 (4)	0.7704 (2)	0.63421 (19)	0.0399 (7)	
C12	0.9602 (4)	0.8536 (2)	0.68244 (19)	0.0420 (7)	
H12A	1.0130	0.8986	0.6451	0.050*	
H12B	1.0649	0.8374	0.7204	0.050*	
C13	1.0396 (5)	0.9900 (3)	0.8188 (2)	0.0537 (9)	
H13A	1.1285	0.9418	0.8042	0.081*	
H13B	1.0520	1.0026	0.8762	0.081*	
H13C	1.0724	1.0434	0.7881	0.081*	
C14	0.3936 (7)	1.0248 (3)	0.6061 (3)	0.0832 (15)	
H14A	0.3917	1.0760	0.5699	0.125*	
H14B	0.3345	1.0413	0.6574	0.125*	
H14C	0.3200	0.9759	0.5821	0.125*	
C15	0.6966 (8)	0.9745 (3)	0.5362 (3)	0.0758 (13)	
H15A	0.8355	0.9615	0.5419	0.114*	
H15B	0.6796	1.0261	0.5011	0.114*	
H15C	0.6304	0.9231	0.5128	0.114*	
C16	0.6372 (7)	0.6755 (2)	0.5647 (2)	0.0584 (10)	
H16A	0.4981	0.6768	0.5520	0.088*	
H16B	0.6662	0.6226	0.5967	0.088*	
H16C	0.7126	0.6738	0.5149	0.088*	
Br1	0.50492 (5)	0.83972 (3)	0.86363 (2)	0.05453 (12)	
Br2	0.94858 (5)	0.78265 (3)	0.86827 (3)	0.06271 (13)	
O	1.0289 (4)	0.71769 (17)	0.61207 (15)	0.0604 (6)	

### Atomic displacement parameters (Å^2^)

**Table:**

	*U*^11^	*U*^22^	*U*^33^	*U*^12^	*U*^13^	*U*^23^
C1	0.0291 (15)	0.0354 (17)	0.0390 (16)	0.0008 (13)	0.0031 (12)	−0.0040 (14)
C2	0.0293 (14)	0.0415 (18)	0.0434 (17)	0.0048 (12)	0.0006 (12)	−0.0004 (15)
C3	0.0355 (15)	0.0398 (17)	0.0391 (16)	−0.0008 (14)	0.0006 (13)	−0.0075 (14)
C4	0.0517 (19)	0.0401 (18)	0.0456 (18)	0.0045 (16)	0.0033 (15)	−0.0135 (16)
C5	0.070 (2)	0.0361 (19)	0.057 (2)	0.0011 (18)	−0.0057 (19)	−0.0068 (18)
C6	0.078 (3)	0.045 (2)	0.067 (3)	−0.006 (2)	−0.003 (2)	0.002 (2)
C7	0.0458 (16)	0.0382 (16)	0.0393 (17)	−0.0022 (14)	−0.0020 (14)	0.0005 (15)
C8	0.0297 (14)	0.0371 (17)	0.0393 (15)	−0.0010 (13)	0.0013 (12)	−0.0028 (14)
C9	0.0397 (14)	0.0435 (17)	0.0419 (16)	−0.0081 (13)	−0.0024 (15)	−0.0010 (16)
C10	0.0522 (18)	0.0337 (16)	0.0316 (15)	−0.0068 (14)	0.0025 (13)	0.0004 (13)
C11	0.0483 (16)	0.0338 (16)	0.0376 (15)	0.0066 (14)	0.0120 (15)	0.0031 (15)
C12	0.0323 (15)	0.0471 (18)	0.0465 (17)	0.0037 (14)	0.0048 (13)	−0.0093 (15)
C13	0.0470 (19)	0.058 (2)	0.0559 (19)	−0.0065 (18)	−0.0019 (16)	−0.0152 (18)
C14	0.069 (3)	0.079 (3)	0.102 (4)	0.008 (2)	−0.009 (2)	0.042 (3)
C15	0.095 (3)	0.072 (3)	0.061 (2)	−0.010 (3)	0.012 (2)	0.009 (2)
C16	0.086 (3)	0.037 (2)	0.052 (2)	−0.009 (2)	−0.0035 (19)	0.0003 (17)
Br1	0.04425 (17)	0.0613 (2)	0.0581 (2)	−0.00202 (17)	0.01213 (17)	0.00965 (18)
Br2	0.0559 (2)	0.0613 (2)	0.0710 (2)	0.01579 (18)	−0.00973 (19)	0.0117 (2)
O	0.0663 (16)	0.0493 (14)	0.0657 (14)	0.0148 (14)	0.0091 (13)	−0.0101 (12)

### Geometric parameters (Å, º)

**Table:**

C1—C2	1.518 (4)	C8—H8	0.9800
C1—C12	1.520 (4)	C9—C10	1.341 (5)
C1—C8	1.524 (4)	C9—H9	0.9300
C1—C3	1.537 (4)	C10—C11	1.470 (4)
C2—C3	1.503 (5)	C10—C16	1.499 (5)
C2—Br2	1.928 (3)	C11—O	1.216 (4)
C2—Br1	1.943 (3)	C11—C12	1.512 (5)
C3—C13	1.518 (4)	C12—H12A	0.9700
C3—C4	1.522 (5)	C12—H12B	0.9700
C4—C5	1.526 (5)	C13—H13A	0.9600
C4—H4A	0.9700	C13—H13B	0.9600
C4—H4B	0.9700	C13—H13C	0.9600
C5—C6	1.517 (5)	C14—H14A	0.9600
C5—H5A	0.9700	C14—H14B	0.9600
C5—H5B	0.9700	C14—H14C	0.9600
C6—C7	1.541 (5)	C15—H15A	0.9600
C6—H6A	0.9700	C15—H15B	0.9600
C6—H6B	0.9700	C15—H15C	0.9600
C7—C14	1.526 (5)	C16—H16A	0.9600
C7—C15	1.529 (5)	C16—H16B	0.9600
C7—C8	1.587 (5)	C16—H16C	0.9600
C8—C9	1.505 (4)		
			
C2—C1—C12	117.1 (3)	C1—C8—C7	115.0 (2)
C2—C1—C8	118.9 (2)	C9—C8—H8	106.6
C12—C1—C8	113.2 (2)	C1—C8—H8	106.6
C2—C1—C3	58.9 (2)	C7—C8—H8	106.6
C12—C1—C3	120.6 (3)	C10—C9—C8	125.7 (3)
C8—C1—C3	117.9 (3)	C10—C9—H9	117.2
C3—C2—C1	61.1 (2)	C8—C9—H9	117.2
C3—C2—Br2	120.5 (2)	C9—C10—C11	119.9 (3)
C1—C2—Br2	120.9 (2)	C9—C10—C16	122.8 (3)
C3—C2—Br1	121.3 (2)	C11—C10—C16	117.2 (3)
C1—C2—Br1	120.8 (2)	O—C11—C10	122.2 (3)
Br2—C2—Br1	106.75 (16)	O—C11—C12	119.3 (3)
C2—C3—C13	118.5 (3)	C10—C11—C12	118.4 (3)
C2—C3—C4	118.8 (3)	C11—C12—C1	113.1 (3)
C13—C3—C4	113.8 (3)	C11—C12—H12A	109.0
C2—C3—C1	59.9 (2)	C1—C12—H12A	109.0
C13—C3—C1	119.3 (3)	C11—C12—H12B	109.0
C4—C3—C1	116.4 (3)	C1—C12—H12B	109.0
C3—C4—C5	113.0 (3)	H12A—C12—H12B	107.8
C3—C4—H4A	109.0	C3—C13—H13A	109.5
C5—C4—H4A	109.0	C3—C13—H13B	109.5
C3—C4—H4B	109.0	H13A—C13—H13B	109.5
C5—C4—H4B	109.0	C3—C13—H13C	109.5
H4A—C4—H4B	107.8	H13A—C13—H13C	109.5
C6—C5—C4	116.4 (3)	H13B—C13—H13C	109.5
C6—C5—H5A	108.2	C7—C14—H14A	109.5
C4—C5—H5A	108.2	C7—C14—H14B	109.5
C6—C5—H5B	108.2	H14A—C14—H14B	109.5
C4—C5—H5B	108.2	C7—C14—H14C	109.5
H5A—C5—H5B	107.4	H14A—C14—H14C	109.5
C5—C6—C7	119.8 (4)	H14B—C14—H14C	109.5
C5—C6—H6A	107.4	C7—C15—H15A	109.5
C7—C6—H6A	107.4	C7—C15—H15B	109.5
C5—C6—H6B	107.4	H15A—C15—H15B	109.5
C7—C6—H6B	107.4	C7—C15—H15C	109.5
H6A—C6—H6B	106.9	H15A—C15—H15C	109.5
C14—C7—C15	106.9 (3)	H15B—C15—H15C	109.5
C14—C7—C6	111.5 (3)	C10—C16—H16A	109.5
C15—C7—C6	106.4 (3)	C10—C16—H16B	109.5
C14—C7—C8	107.6 (3)	H16A—C16—H16B	109.5
C15—C7—C8	112.7 (3)	C10—C16—H16C	109.5
C6—C7—C8	111.7 (3)	H16A—C16—H16C	109.5
C9—C8—C1	109.3 (3)	H16B—C16—H16C	109.5
C9—C8—C7	112.1 (2)		
